# Innate Immune Responses Induced by H9N2 Influenza A Virus and *Klebsiella pneumoniae* Co-Infection

**DOI:** 10.3390/v18080900

**Published:** 2026-08-14

**Authors:** Yong-Jie Zhu, Rui-Rui Du, Feng Xiao, Ling-Yi Shao, Jia-Xin Sun, Yu-Jun Zhou, Yu-Xin Shi, Ya-Wen Jin, Zhi-Jing Xie

**Affiliations:** 1 Shandong Provincial Key Laboratory of Zoonoses, Shandong Agricultural University, Taian 271017, China; 2022110408@sdau.edu.cn (Y.-J.Z.); 2024120665@sdau.edu.cn (R.-R.D.); 2020110477@sdau.edu.cn (F.X.); 2024120703@sdau.edu.cn (L.-Y.S.); 2024120710@sdau.edu.cn (J.-X.S.); 2025121592@sdau.edu.cn (Y.-J.Z.); 2025121528@sdau.edu.cn (Y.-X.S.); 2025121492@sdau.edu.cn (Y.-W.J.); 2 College of Veterinary Medicine, Shandong Agricultural University, Taian 271017, China

**Keywords:** *K. pneumoniae*, H9N2 influenza A virus, transcriptomics, co-infection

## Abstract

*Klebsiella pneumoniae* infection following H9N2 Influenza A virus (IAV) infection causes severe pneumonia. But the underlying pathogenic mechanisms of H9N2 IAV and *K. pneumoniae* co-infection are complex and need to be further explored. In this study, the lung transcriptomes of mice with H9N2 IAV and *K. pneumoniae* co-infection were characterized by transcriptomic profiling. As a result, GO enrichment analysis revealed that the differential genes were primarily involved in the activation of immune responses, cellular components of membranes and extracellular spaces, and defense responses against pathogen infections. According to KEGG enrichment, the differentially expressed genes (DEGs) were concentrated in TLR signaling pathways, RLR signaling pathways, TNF signaling pathways and NLRP3 signaling pathways. Furthermore, in vitro cell models were established to investigate the innate immune responses induced by H9N2 IAV and *K. pneumoniae* CPS co-stimulation. *K. pneumoniae* CPS stimulation influenced the cytokine profiles of mink lung epithelial cells infected with H9N2 IAV, worsened cell viability, and aggravated apoptosis, indirectly inhibiting H9N2 IAV replication. The findings demonstrated that *K. pneumoniae* superinfection modulated the innate immune responses induced by H9N2 IAV infection, contributing to its pathogenesis.

## 1. Introduction

Respiratory diseases are very frequent in both humans and animals worldwide, causing high morbidity and mortality with an economic burden on healthcare systems [1,2,3,4]. Influenza A virus (IAV) is one of the prevalent and serious respiratory pathogens causing respiratory infections in humans and animals. Bacterial superinfections following IAV infections, an underlying etiology of community and hospital-acquired infections, exacerbate diseases with increased morbidity and mortality during IAV epidemics [5,6]. The innate immune system of the respiratory tract can immediately eliminate bacterial infections during homeostasis [7]. But IAV infection can cause respiratory physical barrier lesions, immune cell dysfunction, and severe hyperinflammatory responses, contributing to a favorable pulmonary environment for bacterial superinfections [8,9,10]. However, detailed knowledge of IAV and bacterial co-infections needs to be clarified, which will ultimately facilitate more effective approaches and strategies for the prevention and treatment of respiratory diseases caused by IAV and bacterial co-infections.

IAVs are divided into subtypes according to the combination of hemagglutinin (HA) and neuraminidase (NA), and 19 HA and 11 NA subtypes have been identified among IAVs [11,12]. Many studies have focused on H5N1 and H7N9 IAVs due to their highly pathogenic phenotype in poultry, with a pressing public health concern [11,13]. H9N2 IAV belongs to low-pathogenic IAV and its infection causes mild respiratory disease without priority for animal disease control in many countries [11,13,14]. Indeed, H9N2 IAVs widely circulate in poultry, wild birds and mammals, which have been associated with cases of zoonotic infections, posing a significant threat to global health [11,13,15]. Furthermore, cases with H9N2 IAV infection are highly susceptible to bacterial superinfections, with severe respiratory disease and mortality. Broilers with co-infection of *Ornithobacterium rhinotracheale* (*O. rhinotracheale*) and H9N2 IAV exhibited severe pneumonia [16]. H9N2 IAV infection predisposed to secondary *Pseudomonas aeruginosa* (*P. aeruginosa*) infection and facilitated the development of severe pneumonia in mink [17]. Co-infection with H9N2 IAV and *Escherichia coli* contributed to the development of severe lung injury in BALB/c mice [18].

*K. pneumoniae* is a Gram-negative opportunistic bacterium and is widely present in the environment and settled in many mammals, where it is responsible for a variety of community and hospital infections worldwide, constituting one of the most formidable threats to global healthcare [19,20]. Capsular polysaccharides (CPS) are one of the main factors that make bacteria highly virulent, and their structure and composition can affect the virulence of pneumonia-causing *K. pneumoniae*. They mainly help the pathogen evade the immune system during infection by protecting the bacteria from being engulfed and destroyed by serum, promoting the survival, colonization, and spread of the pneumonia-causing bacteria [21]. Based on variations in the capsule constituents and structures, *K. pneumoniae* was classified into at least 79 chemically distinct serotypes by the traditional serotyping method [16,21]. *K. pneumoniae* serotype K2 is one of the most prevalent serotypes and is frequently reported in community-acquired pneumonia [22,23,24]. *K. pneumoniae* serotype K2 is a growing threat to mink, livestock and poultry [25]. *K. pneumoniae* might be a commensal organism that only causes disease in certain circumstances. H9N2 IAV infection facilitated secondary *K. pneumoniae* infection, causing severe pneumonia [26]. But the underlying pathogenic mechanisms of H9N2 IAV and *K. pneumoniae* co-infection are complex and need to be further explored.

Transcriptomic profiling provides a comprehensive view of gene expression dynamics, offering distinct advantages in screening specific genes that influence host–pathogen interactions and elucidating pathogenic mechanisms [27,28,29]. In this study, the lung transcriptomes of mice with H9N2 IAV and *K. pneumoniae* co-infection were characterized by transcriptomic profiling. Furthermore, in vitro lung epithelial cell models were established to investigate the innate immune responses against stimulation with *K. pneumoniae* CPS following H9N2 IAV infection. Different molecular signatures were screened between H9N2 IAV mono-infection, *K. pneumoniae* mono-infection, and H9N2 IAV and *K. pneumoniae* co-infection, contributing to the prevention and control of H9N2 IAV and *K. pneumoniae* co-infection.

## 2. Materials and Methods

### 2.1. H9N2 IAV and K. pneumoniae

Based on previous studies on the co-infection of H9N2 IAV and *K. pneumoniae* [26], A/mink/Shandong/F10/2013 (H9N2) (Mk/SD/F10/13) and *K. pneumoniae*-SD-21 were selected for this study. Mk/SD/F10/13 was isolated from the mink showing respiratory symptoms, belonging to Y280-like sublineage [14]. *K. pneumoniae*-SD-21 was isolated from the mink exhibiting pneumonia and belongs to serotype K2 with hypermucoviscosity phenotype [25]. Both isolates were preserved in our laboratory.

### 2.2. Co-Infection Experiment Designs

**Figure 1:**
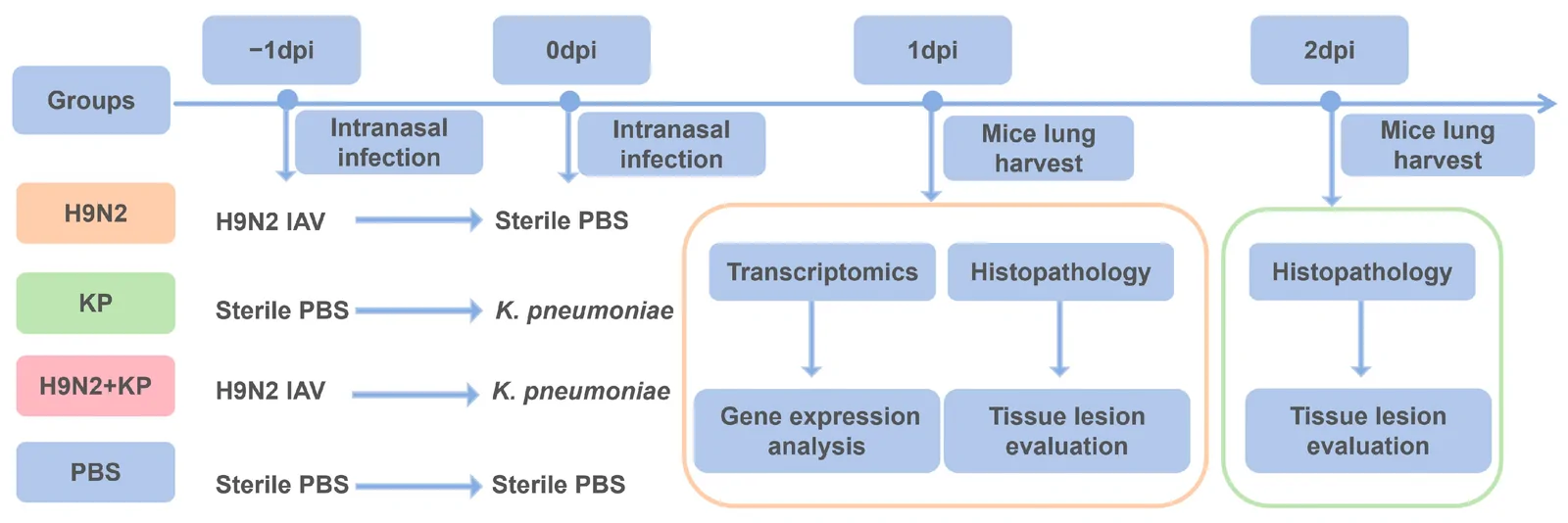
Schematic diagram of the animal experimental design.

Animal experiments were performed using Kunming (KM) mice via a modified method based on a previous report [26]. In brief, 80 KM mice were randomly divided into four equal groups. The mice were intranasally challenged with Mk/SD/F10/13 and/or *K. pneumoniae.* The day of *K. pneumoniae* inoculation was designated as day 0 post-infection (0 dpi). The mice in Group 1 (H9N2 group) were inoculated with 3.6 × 10^7.0^ EID_50_ Mk/SD/F10/13 at −1 dpi and sterile PBS at 0 dpi, Group 2 (KP group) with 5 × 10^3.0^ CFU *K. pneumoniae* on 0 dpi and sterile PBS at −1 dpi, Group 3 (H9N2+KP group) with 5 × 10^3.0^ CFU *K. pneumoniae* at 0 dpi and 3.6 × 10^7.0^ EID_50_ Mk/SD/F10/13 at −1 dpi, and Group 4 (PBS group) with sterile PBS twice at a 1-day interval. The schematic diagram of the animal experimental design is shown in Figure 1.

### 2.3. Clinical Examination

Mice were monitored daily to record changes in general physical condition, and a 0–3 clinical scoring scale was used to assess depression-like signs: score 0 represents normal activity, sleek fur and normal appetite; score 1 refers to slightly reduced movement and mildly ruffled fur; score 2 indicates obvious lethargy, decreased food intake and huddling behavior; score 3 stands for severe inactivity, persistent listlessness and marked anorexia.

### 2.4. Histopathologic Lesions

Histopathologic lesions in lungs were assayed as described in the previous report [6]. In brief, 3 mice in each group were euthanized and weighed at 24 and 48 h post-infection (hpi), respectively. The lungs were sampled and weighed. Parts of the lung tissues were used for histopathologic assays.

### 2.5. Transcriptomics Assays

To perform transcriptomics assays, three mice in each group were euthanized on 1 dpi, respectively. Parts of the lungs were rapidly sampled for transcriptome assays, referring to previous reports [27,28]. In brief, total RNA was extracted from the tissues, and the purity, concentration, and integrity of the extracted RNA were assessed. Libraries were constructed and subjected to quality control. Following successful library quality control, transcriptome sequencing was performed using a high-throughput sequencing platform. Raw data underwent the following filtering: removal of adapter sequences; exclusion of low-quality sequences (including those with >10% N-substitutions and those with >50% bases having Q-score ≤ 10). The mice in the H9N2 group, KP group, and H9N2+KP group were compared against the mice in PBS group. Differential gene expression screening was performed using the DESeq2 differential analysis software from the R package. Thresholds of log2FoldChange > 1 or <−1 and *p* < 0.05 were set to identify upregulated and downregulated genes. Transcriptomic results were validated via qPCR in conjunction with transcriptomics findings and co-infection pathogenesis mechanisms.

### 2.6. In Vitro Cell Models Co-Stimulation with H9N2 IAV and K. pneumoniae CPS

**Figure 2:**
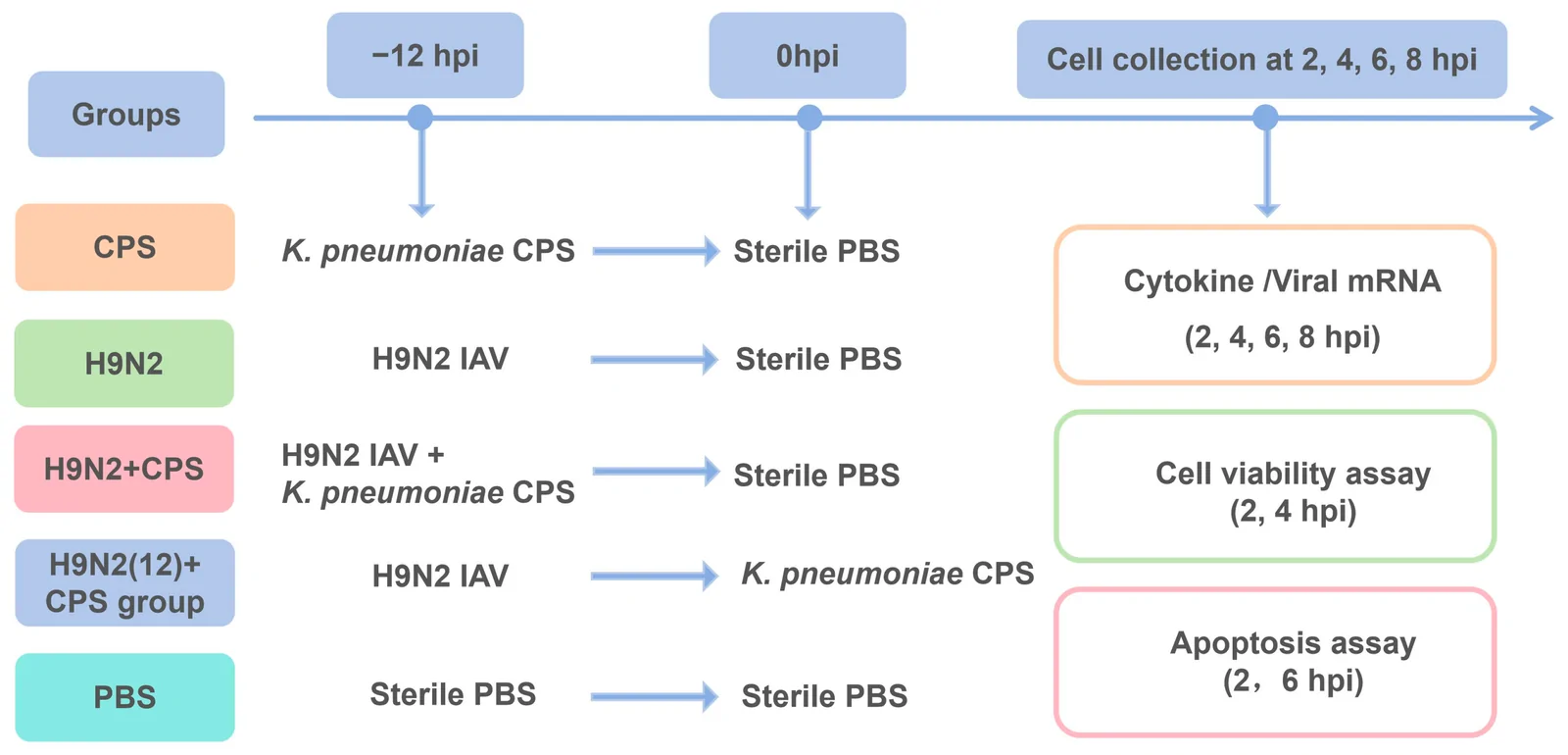
Schematic diagram of the in vitro cell experimental design.

Based on the transcriptomics analysis, in vitro cell models with H9N2 IAV and *K. pneumoniae* CPS co-stimulation were established. *K. pneumoniae* CPS was extracted and purified by a modified method, referring to a previous report [30]. Mv.1.Lu cells (mink lung epithelial cell line) were kindly provided by the Laboratory of Biological Products, Shandong Agricultural University, and cultured at 37 °C in a 5% CO_2_ incubator. In the experiment, 0 hour post-infection (0 hpi) was defined as the time when group 4 received *K. pneumoniae* CPS stimulation after H9N2 IAV infection. Group 1 (CPS group) received CPS at 10 μg/mL at −12 hpi and PBS at 0 hpi, Group 2 (H9N2 group) was treated with Mk/SD/F10/13 (MOI = 1) at −12 hpi and PBS at 0 hpi, Group 3 (H9N2+CPS group) was treated with Mk/SD/F10/13 (MOI = 1) and CPS at 10 μg/mL at −12 hpi, Group 4 (H9N2(12)+CPS group) was treated with Mk/SD/F10/13 (MOI = 1) at −12 hpi and CPS at 10 μg/mL at 0 hpi, and Group 5 (PBS group) was treated with PBS at −12 hpi and 0 hpi. The schematic diagram of the in vitro cell experimental design is shown in Figure 2.

To investigate the impact of *K. pneumoniae* CPS stimulation on inflammatory responses following H9N2 IAV infection, the mRNA expression levels of *Tnf*, *Ifng* and *Il4* were evaluated in Mv1Lu mink lung epithelial cells at 2, 4, 6, and 8 hpi using qRT-PCR. The primers for *Tnf*, *Ifng* and *GAPDH* referred to a previous study [31], and *Il4* referred to a previous study [32]. Fold change ratios relative to control samples were calculated for each gene and normalized to *GAPDH* using the ΔΔCt method [33]. To explore the influences of *K. pneumoniae* CPS stimulation following H9N2 IAV infection on the viability of the lung epithelial cells, cell viability at 2 and 4 hpi was detected, and the optical density (OD) was determined at 450 nm. Cell apoptosis was observed at 2 hpi and 6 hpi. The apoptotic cell percentage was determined as the ratio of apoptotic cells to total cells multiplied by 100%. To determine whether *K. pneumoniae* CPS stimulation influences H9N2 IAV replication in the lung epithelial cells, the HA gene segments of H9N2 IAV were detected at 2, 4, 6 and 8 hpi, using qRT-PCR as previously described [34].

### 2.7. Statistical Analysis

All data were expressed as mean ± SEM and statistically analyzed using SPSS version 27.0 (IBM Corporation, Armonk, NY, USA). After confirming the normal distribution of the data using the Shapiro–Wilk test, one-way analysis of variance (ANOVA) was conducted, with Duncan’s multiple range test applied as the post hoc multiple-comparison procedure following ANOVA. GraphPad Prism 8.0 software (GraphPad Software Inc., La Jolla, CA, USA) was used for data visualization.

## 3. Results

### 3.1. K. pneumoniae Superinfection Following H9N2 IAV Infection Exacerbated Pulmonary Histopathological Lesions

**Figure 3:**
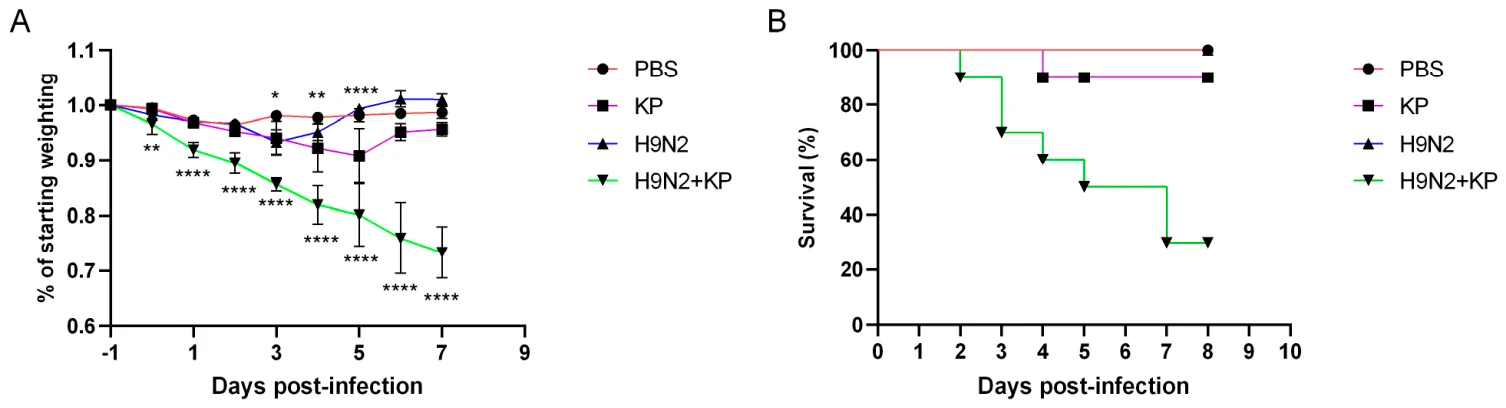
Clinical changes in mice within 7 days post-infection. (**A**) Dynamic changes in body weight (% of starting weighting); (**B**) Survival (%). In Figure 3B, the survival rates of both the H9N2 group and the PBS group were 100%, and therefore the survival curves of the two groups overlapped. Significance: * *p* < 0.05, ** *p* < 0.01, **** *p* < 0.0001.

**Figure 4:**
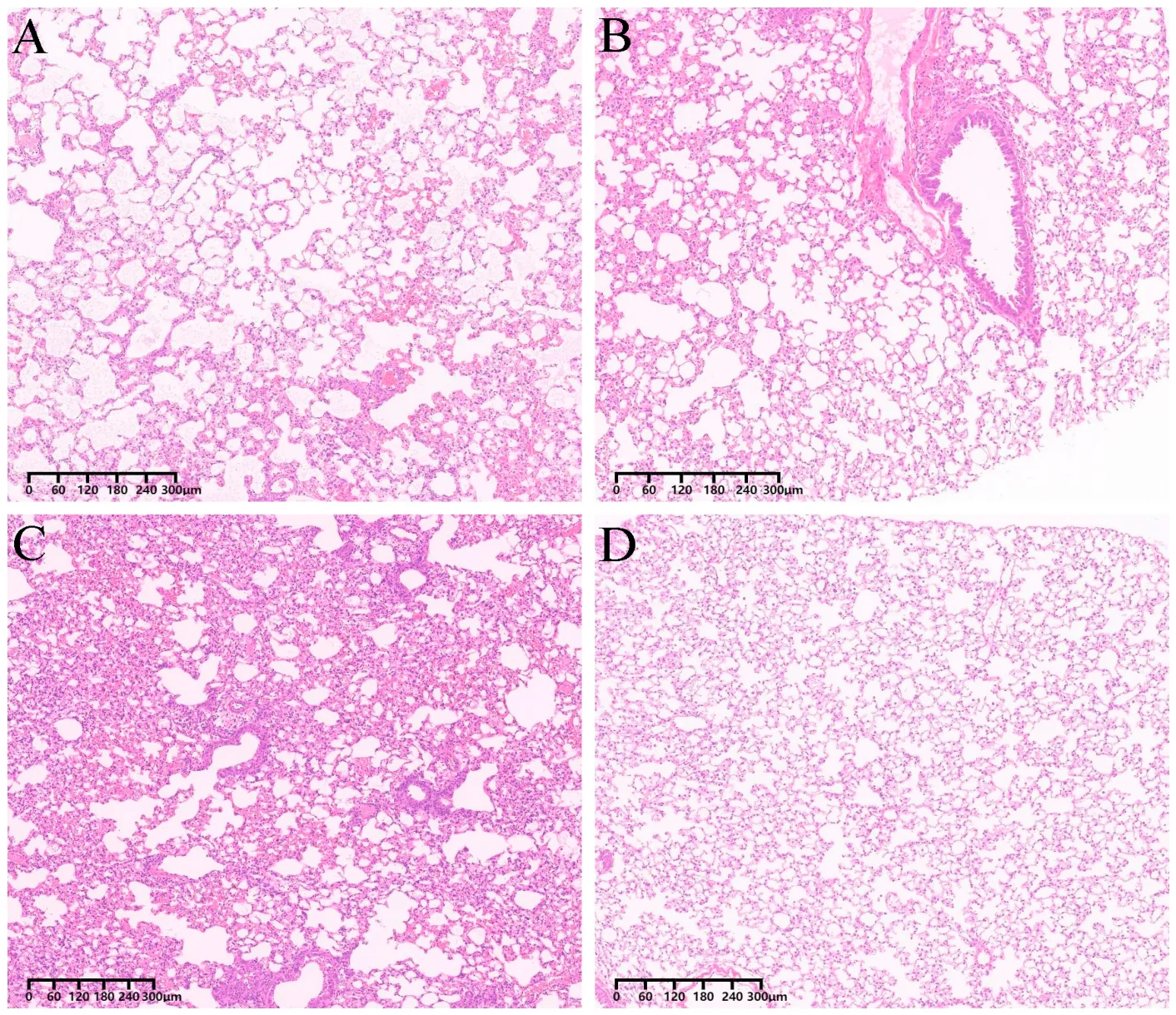
Lung histopathological lesions of the mice (HE, ×200). (**A**) H9N2 group, (**B**) KP group; (**C**) H9N2+KP group; (**D**) PBS group.

At 24 hpi, the mice in the PBS group showed no obvious clinical signs. The mice in the H9N2 group showed slight inappetence. The mice in the KP group exhibited signs of depression, reduced appetite, and decreased activity. However, mice in the H9N2+KP group displayed anorexia, weakness, unkempt fur, and a curled body posture, and mice continued to die (Figure 3A,B). The representative histopathological lesions of the lungs sampled from the experimental mice at 24 hpi are shown in Figure 4. In the H9N2 group, the lungs of the mice exhibited vascular dilation and hemorrhage, extensive alveolar cavity exudate, a thickened alveolar interstitium, and inflammatory cell infiltration, and representative pathological lesions are presented in Figure 4A. A thickened pulmonary interstitium and infiltration of inflammatory cells were found in the lungs of the mice in the KP group, with representative lesions illustrated in Figure 4B. Secondary *K. pneumoniae* following H9N2 IAV infection caused more severe pathological lesions including marked vascular dilation and hemorrhage, subvascular interstitial edema with fluid exudation, a thickened alveolar interstitium, more significant infiltration of numerous inflammatory cells, and alveolar atrophy and consolidation, and representative histopathological lesions are displayed in Figure 4C. No histopathological lesions were observed in the lungs of the mice in PBS group, as shown in Figure 4D. The findings implied that co-infection with H9N2 IAV and *K. pneumoniae* promoted the development of the disease.

### 3.2. Transcriptomic Analysis of the Lungs of the Experiment Mice

**Figure 5:**
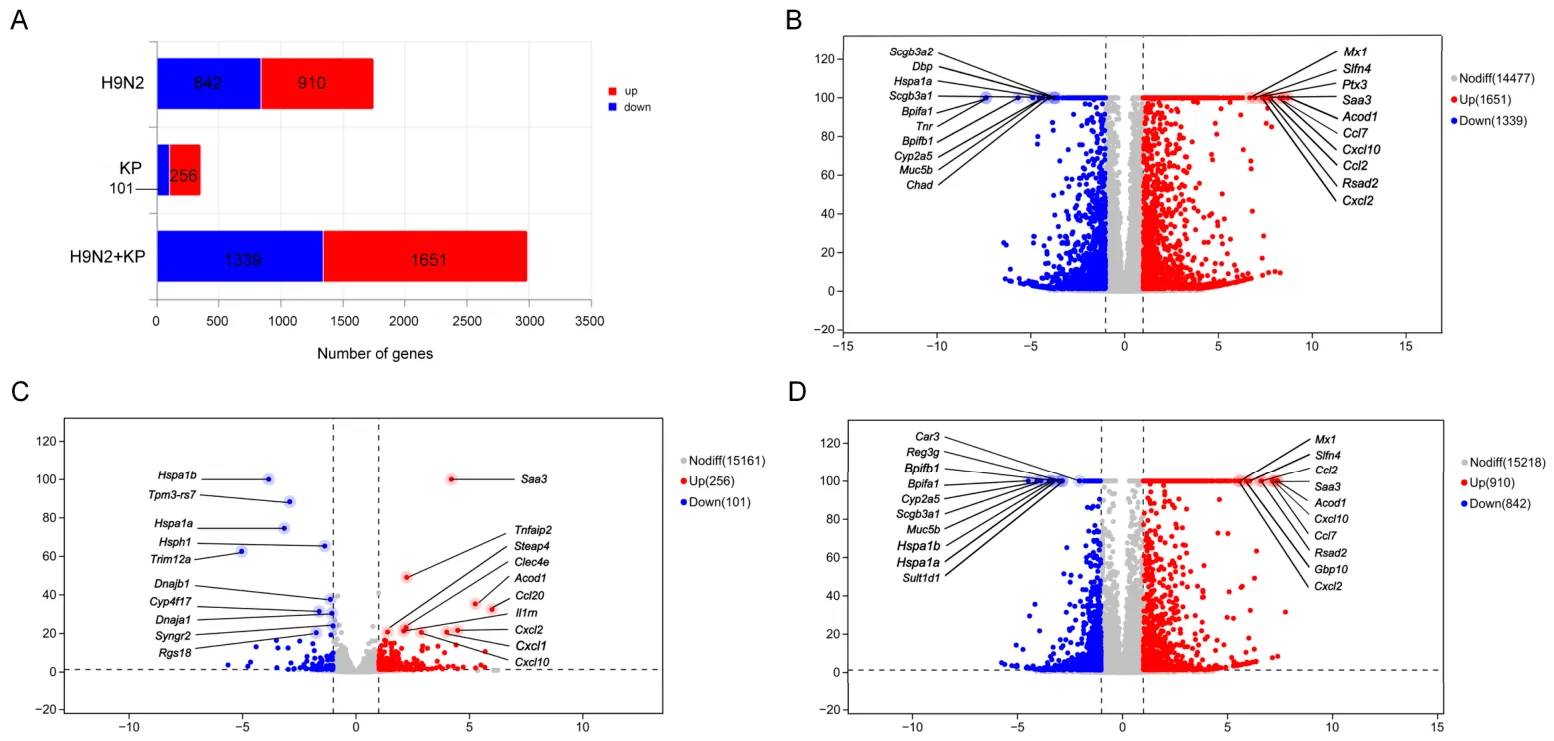
Transcriptomic profiles and volcano maps. (**A**) Transcriptomic profiles; (**B**) H9N2+KP group; (**C**) KP group; (**D**) H9N2 group. The y-axis represents the *p* value for gene count differences, and the x-axis displays the Log2Fold Change. Upregulated genes are marked by red dots, downregulated genes by blue dots, and insignificant genes (*p* < 0.05) by gray dots.

**Figure 6:**
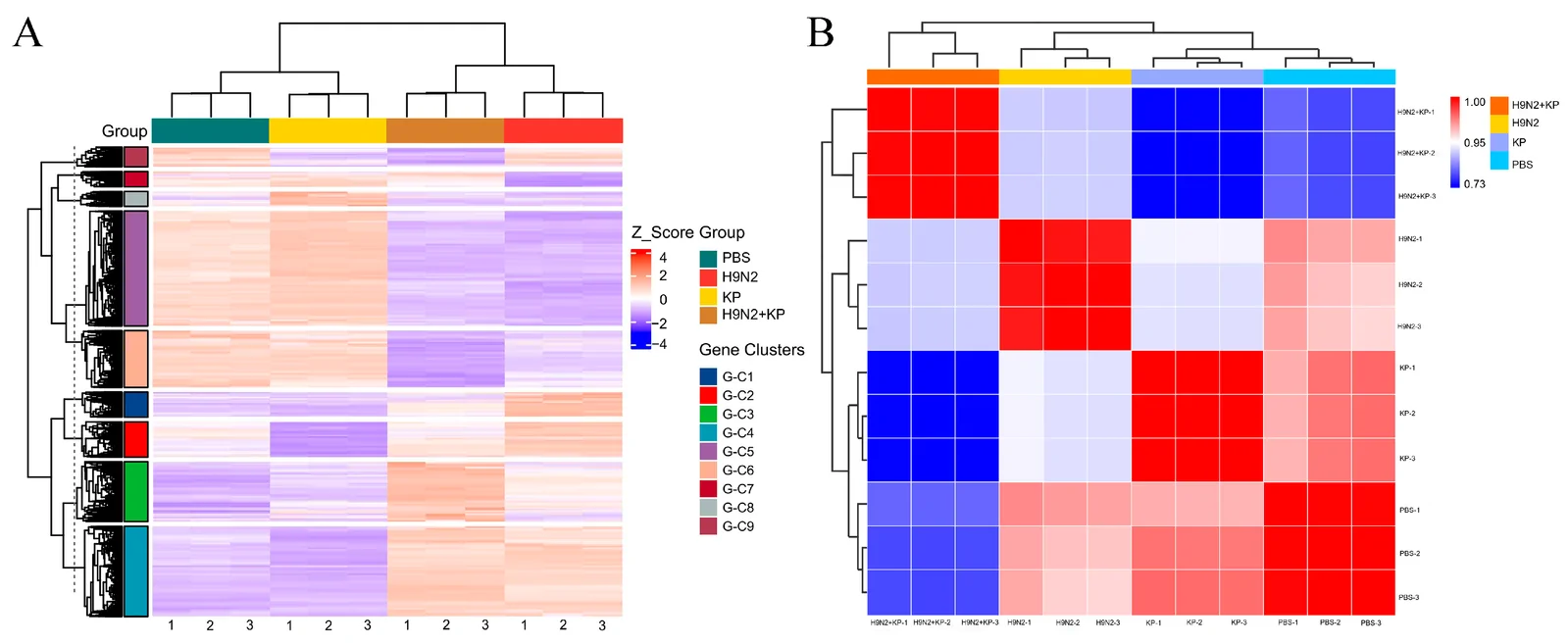
Sample correlations. (**A**) Differential gene clustering analysis heatmaps; (**B**) Correlation analysis heatmaps. The color bar on the right shows the expression level: the brighter the red, the higher the expression; the brighter the blue, the lower the expression. The vertical dotted line on the left demarcates boundaries of distinct gene clusters defined by hierarchical clustering.

Compared with the PBS group, there were 2990 differential genes in the H9N2+KP group including 1651 upregulated genes and 1339 downregulated genes, 1752 differential genes in the H9N2 group, including 910 upregulated genes and 842 downregulated genes, 357 genes in the KP group, including 256 upregulated genes and 101 downregulated genes (Figure 5A). Significant differences in gene expression were observed among the H9N2 group, the KP group, and the H9N2+KP group. The volcano plots indicated significant differences with a significance threshold of *p* < 0.05 (Figure 5B–D). The heatmaps showed the gene differences among the four groups, with greater inter-group gene differences and smaller intra-group gene differences (Figure 6).

**Figure 7:**
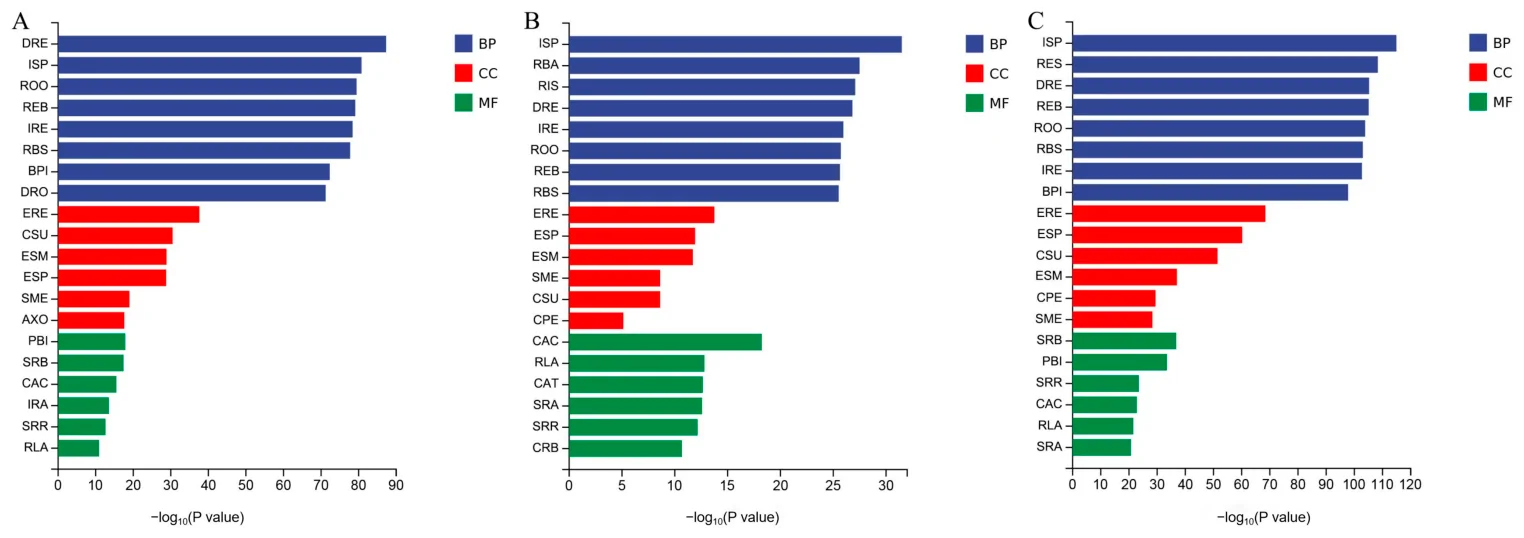
GO enrichment. (**A**) H9N2 group; (**B**) KP group; (**C**) H9N2+KP group. AXO, Axoneme; BPI, Biological process involved in interspecies interaction between organisms; CAC, Cytokine activity; CAT, Chemokine activity; CPE, Cell periphery; CRB, Chemokine receptor binding; CSU, Cell surface; DRE, Defense response; DRO, Defense response to other organism; ERE, Extracellular region; ESM, External side of plasma membrane; ESP, Extracellular space; IRA, Immune receptor activity; IRE, Immune response; ISP, Immune system process; PBI, Protein binding; RBA, Response to bacterium; REB, Response to external biotic stimulus; RES, Response to external stimulus; RIS, Regulation of immune system process; RLA, Receptor ligand activity; ROO, Response to other organism; RBS, Response to biotic stimulus; SME, Side of membrane; SRB, Signaling receptor binding; SRR, Signaling receptor regulator activity; SRA, Signaling receptor activator activity.

**Figure 8:**
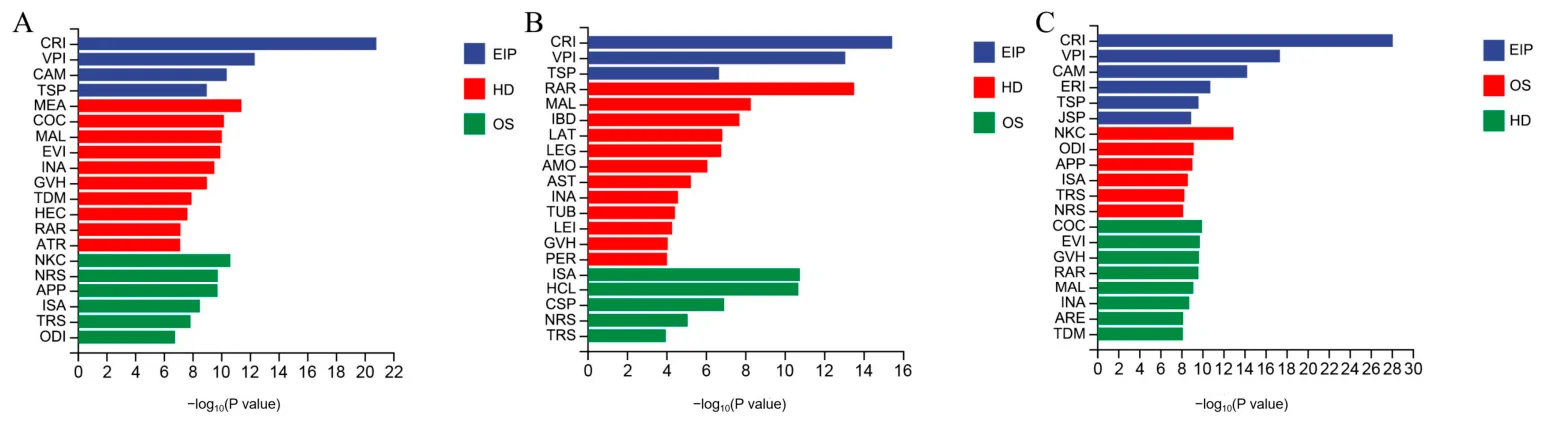
KEGG enrichment. (**A**) H9N2 group; (**B**) KP group; (**C**) H9N2+KP group. AMO, Amoebiasis; APP, Antigen processing and presentation; ARE, Allograft rejection; AST, Asthma; ATR, African trypanosomiasis; CAM, Cell adhesion molecules; CRI, Cytokine-cytokine receptor interaction; COC, Coronavirus disease-COVID-19; CSP, Chemokine signaling pathway; ERI, ECM-receptor interaction; EVI, Epstein–Barr virus infection; GVH, Graft-versus-host disease; HCL, Hematopoietic cell lineage; HEC, Hepatitis C; IBD, Inflammatory bowel disease; INA, Influenza A; ISA, IL-17 signaling pathway; JSP, JAK-STAT signaling pathway; LAT, Lipid and atherosclerosis; LEG, Legionellosis; LEI, Leishmaniasis; MAL, Malaria; MEA, Measles; NKC, Natural killer cell mediated cytotoxicity; NRS, NOD-like receptor signaling pathway; ODI, Osteoclast differentiation; PER, Pertussis; RAR, Rheumatoid arthritis; TDM, Type I diabetes mellitus; TRS, Toll-like receptor signaling pathway; TSP, TNF signaling pathway; TUB, Tuberculosis; VPI, Viral protein interaction with cytokine and cytokine receptor; EIP, Environmental information processing; HD, Human diseases; OS, Organismal systems.

GO enrichment analysis revealed that the upregulated genes in the H9N2 group, KP group, and H9N2+KP group were more involved in the activation of immune responses, molecular activation related to signaling pathways, cellular components of membranes and extracellular spaces, and defense responses against pathogen infections (Figure 7). It demonstrated that the immune responses varied among the different infection combinations of H9N2 IAV and *K. pneumoniae*. In the mice with H9N2 IAV and *K. pneumoniae* co-infection, the genes involved in signaling pathways were highly enriched and activated many cascade reactions, resulting in more intense immunopathological responses. KEGG enrichment analysis showed that many upregulated genes in the H9N2, KP, and H9N2+KP groups were involved in cytokine and cytokine receptor interactions, viral protein-related cytokines and receptors, and the TNF, NLR, IL-17, and TLR signaling pathways (Figure 8). Based on GO enrichment analysis and KEGG enrichment analysis, in the lungs of the mice in the H9N2 group, the important upregulated genes were *tlr2*, *tlr9*, *irf7*, *irf9*, *nlrp3*, etc., and the important downregulated genes included *cyp2e1*, *per3*, *hspa1b*, *dnaja1*, etc. In the lungs of the mice in KP group, the important upregulated genes included *tlr2*, *cd14*, *nlrp3*, *nlrp1b*, and *mefv*, etc., and the important downregulated genes included *hspa1a*, *hspa1b*, *hspb1* and *dnaja1*, etc. In the lungs of the mice in H9N2+KP group, the important upregulated genes were *tlr2*, *tlr9*, *irf9*, *nlrp3*, *oas1*, etc., and the important downregulated genes included *cyp2e1*, *per3*, *hspa1b*, *dnaja1*, etc.

### 3.3. Stimulation of K. pneumoniae CPS Exacerbates the Inflammatory Responses of Mv.1.Lu Cells Infected with H9N2 IAV and Influenced Virus Replication

**Figure 9:**
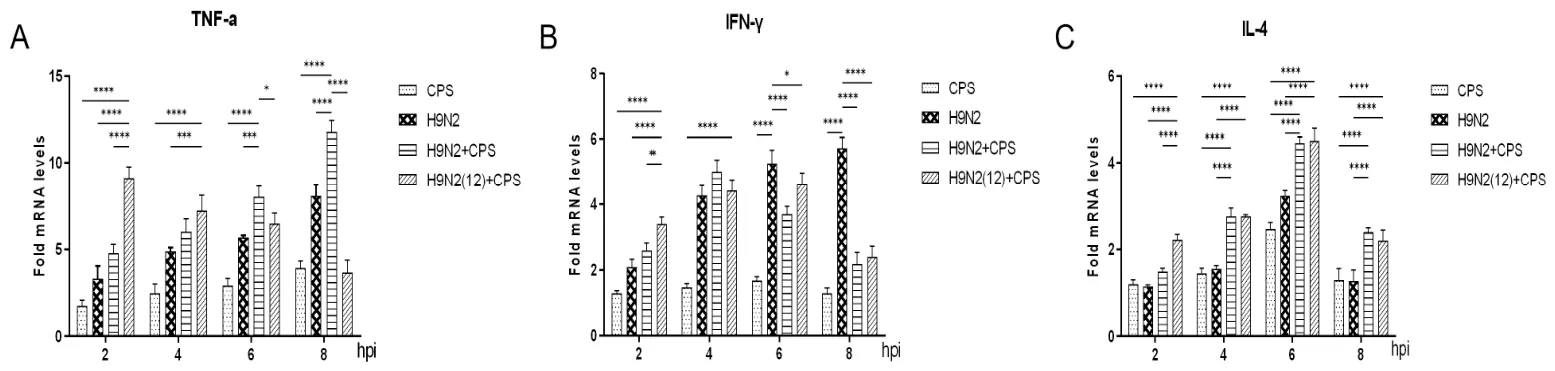
Relative mRNA expression levels of *Tnf*, *Ifng*, and *Il4* following CPS and H9N2 treatment. Relative mRNA expression of (**A**) *Tnf*, (**B**) *Ifng*, (**C**) *Il4* in mink lung epithelial cells from the CPS, H9N2, H9N2+CPS, and H9N2(12)+CPS groups at 2, 4, 6, and 8 hpi as measured by qRT-PCR. Data are presented as mean ± SEM. Significance: * *p* < 0.05, ** *p* < 0.01, *** *p* < 0.001, **** *p* < 0.0001.

As shown in Figure 9A, *Tnf* mRNA levels in the CPS group, H9N2 group and H9N2+CPS group were gradually increased at 2, 4, 6 and 8 hpi, but gradually decreased in the H9N2 (12 h)+CPS group. *Tnf* mRNA levels in the H9N2 (12 h)+CPS group were significantly higher than those in the other groups at 2 hpi (*p* < 0.0001). *Tnf* mRNA levels in the H9N2 (12 h)+CPS group were significantly higher than those in the CPS group and H9N2 group at 4 hpi (*p* < 0.001). *Tnf* mRNA levels in the H9N2+CPS group were significantly higher than those in the other groups at 6 hpi and 8 hpi (*p* < 0.05). *Ifng* mRNA levels in H9N2 (12 h)+CPS group were significantly higher than those in other groups at 2 hpi (*p* < 0.01) (Figure 9B). But *Ifng* mRNA levels in the H9N2+CPS group were significantly higher than those in the other groups at 4 hpi (*p* < 0.0001). *Ifng* mRNA levels in the H9N2 group were significantly higher than those in other groups at 6 and 8 hpi (*p* < 0.05). *Il4* mRNA levels in the H9N2+CPS group and the H9N2 (12 h)+CPS group were significantly higher than those in the CPS group and the H9N2 group at 2, 4, 6 and 8 hpi (*p* < 0.0001) (Figure 9C). This demonstrated that co-stimulation with CPS and H9N2 IAV increased the expression of *Il4*.

**Figure 10:**
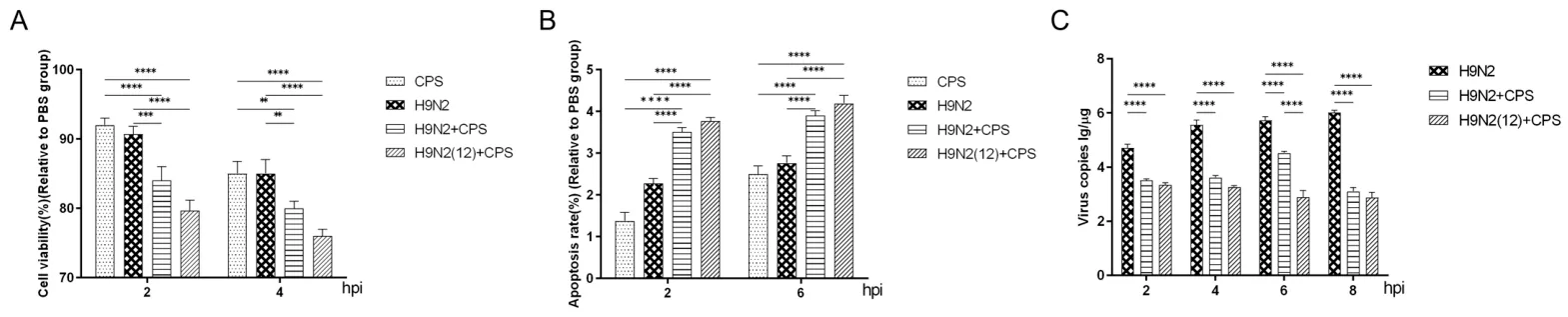
Cell viability, apoptosis, and viral nucleic acid levels. (**A**) Cell viability in the CPS group, H9N2 group, H9N2+CPS group, and H9N2(12)+CPS group at 2 and 4 hpi. (**B**) Apoptosis levels in CPS group, H9N2 group, H9N2+CPS group, and H9N2(12)+CPS group at 2 and 6 hpi. (**C**) The viral nucleic acid levels in H9N2 group, H9N2+CPS group and H9N2(12)+CPS group at 2, 4, 6 and 8 hpi. Significance: ** *p* < 0.01, *** *p* < 0.001, **** *p* < 0.0001.

Compared to the PBS group, the cell viability in the CPS group, H9N2 group, H9N2+CPS group, and H9N2 (12 h)+CPS group showed a downward trend at 2 and 4 hpi, with significant differences (*p* < 0.01) (Figure 10A). And the viability of cells in the H9N2+CPS group and H9N2 (12 h)+CPS group was significantly lower than that in the CPS group and H9N2 group at 2 and 4 hpi (*p* < 0.01). The cell viability in the H9N2 (12 h)+CPS group was lowest at 2 and 4 hpi (*p* < 0.0001). As shown in Figure 10B, the apoptosis levels in the CPS group, H9N2 group, H9N2+CPS group, and H9N2 (12 h)+CPS group exhibited an upward trend over time. The apoptotic cells in the CPS group, H9N2 group, H9N2+CPS group and H9N2 (12 h)+CPS group were significantly more numerous than in the PBS group at 6 hpi (*p* < 0.0001). Compared with the CPS group and H9N2 group, the apoptotic cells in the H9N2+CPS group and H9N2 (12 h)+CPS group were significantly more numerous at 2 and 6 hpi (*p* < 0.0001). At 2, 4, 6 and 8 hpi, the viral nucleic acid levels in the H9N2 group were significantly higher than those in the H9N2+CPS group and H9N2 (12 h)+CPS group (*p* < 0.0001) (Figure 10C), with a significant difference between the H9N2+CPS group and H9N2 (12 h)+CPS group at 6 hpi (*p* < 0.0001). The findings demonstrated that *K. pneumoniae* CPS stimulation influenced the cytokine profiles of the cells infected with H9N2 IAV, contributing to the pathogenesis of *K. pneumoniae* and H9N2 IAV co-infection and affecting H9N2 IAV replication in the cells.

## 4. Discussion

Bacterial superinfections following H9N2 IAV infections are common in both humans and animals worldwide, contributing to the development of respiratory diseases [11,13,15,16,17]. H9N2 IAV infection facilitates secondary *K. pneumoniae* superinfection and triggers severe pneumonia, as IAV impairs antibacterial immune responses and restricts pulmonary bacterial clearance [26,35,36,37]. However, the pathogenic features and host immune mechanisms underlying H9N2 IAV–*K. pneumoniae* co-infection remain incompletely understood. In this study, we investigated pulmonary pathological changes and molecular mechanisms of co-infection using a mouse model, combined with histological examination and transcriptomic profiling.

Co-infection triggered more severe pulmonary inflammatory infiltration, structural disruption, and tissue damage than an H9N2 IAV single infection. GO enrichment analysis revealed that the DEGs were primarily associated with immune response activation, membrane and extracellular cellular components, and defense responses against pathogenic infections. KEGG enrichment analysis further indicated that the DEGs were involved in the TLR, RLR, TNF, and NLRP3 signaling pathways. The TLR pathway mediates pathogen recognition and triggers pro-inflammatory responses, and excessive activation can lead to cytokine storms [35,38]. The RLR pathway senses viral RNA and induces type I interferon antiviral responses but may hinder the clearance of extracellular bacteria [2,9]. The TNF signaling pathway enhances inflammatory cascades and recruits neutrophils, and excessive activity can damage the alveolar-capillary barrier [4,10,17]. The NLRP3 inflammasome mediates the maturation of inflammatory factors and programmed cell death, serving as a key target linking inflammation with lung tissue damage [2,5].

The capsule is an important outermost-layer virulence factor of *K. pneumoniae* and protects bacteria against the host innate immunity [39]. Current CPS research mostly focuses on virulence, synthesis, and vaccine targets in single bacterial infection models, with few reports on its immune regulatory role in virus-bacteria co-infection. Our study revealed that secondary CPS stimulation in H9N2 IAV-infected cells altered the immune profile by significantly upregulating the mRNA levels of pro-inflammatory cytokines *Tnf* and the immunomodulatory cytokine *Il4*, while dynamically regulating *Ifng* levels. Specifically, the marked upregulation of *Tnf* mRNA expression exacerbated the inflammatory response. TNF-α also contributed to inflammation caused by H9N2 IAV and *P. aeruginosa* co-infections [17,33,40]. IFN-γ plays an important role in preventing virus infection and in the promotion of innate and adaptive immune responses [41]. In the late stage of infection, the H9N2 (12 h)+CPS group showed significantly decreased *Tnf* and *Ifng* levels, possibly due to two mechanisms: early strong inflammation triggers immune negative feedback to avoid excessive damage, and severe cell apoptosis causes massive depletion of cytokine-producing epithelial cells. This downward trend suggests a shift from early hyperinflammation to an immunosuppressive phase, which facilitates the persistent presence of pathogens. In addition, at 8 hpi, the *Ifng* mRNA expression in the group infected with H9N2 IAV alone was higher than in the other groups, which may reflect a complete antiviral response in the absence of bacterial co-stimulation. In the co-stimulation group, *Ifng* rapidly declined after peaking, which might be related to CPS-mediated immunosuppression, excessive apoptosis of IFN-γ-producing cells, and inflammatory negative feedback. CPS and H9N2 IAV co-stimulation increased *Il4 mRNA* expression. IL-4 generally mediates anti-inflammatory and humoral immune responses, but excessive IL-4 during co-infection can suppress Th1-type antibacterial/antiviral responses, weakening the host’s ability to clear both pathogens. At the same time, IL-4 induces macrophages to polarize toward the M2 type, facilitating bacterial immune evasion and worsening pathological damage [42]. Lung epithelial cells play a key role in the innate immune responses against *K. pneumoniae* by ingesting and controlling microorganisms and facilitating their opsonization [43]. In this study, co-stimulation with CPS and H9N2 IAV worsened the cell viability and aggravated apoptosis of the cells infected with H9N2 IAV and reduced H9N2 IAV replication in these cells. The mechanism may be that H9N2 relies heavily on structurally intact, metabolically stable epithelial cells to complete its replication cycle, while CPS combined with H9N2 triggers severe inflammatory storms, continuously disrupting cellular homeostasis and metabolism, amplifying damage and apoptosis, and thus hindering viral replication. Similarly, *E. coli* LPS can indirectly inhibit avian influenza virus replication in chicken macrophages [38], and *Pseudomonas aeruginosa* LPS can also indirectly reduce H9N2 replication in mouse alveolar macrophages after H9N2 infection [33]. Further systematic functional investigations both in vitro and in vivo should be performed to elucidate this regulatory pathway. Overall, virus-bacteria co-infection significantly elevates pro-inflammatory cytokine levels, potentially triggering a cytokine storm and leading to severe lung tissue damage.

This study has certain limitations: First, only a single time point for transcriptome analysis was set, so it cannot reflect the dynamics of the co-infection immune response. Second, we only used qRT-PCR to validate the mRNA levels of a subset of genes, and protein-level analyses including Western blot and ELISA were not carried out due to limited remaining samples and a tight project schedule. Third, we only detected viral HA gene copies in mink lung epithelial cells in vitro, and viral burden in mouse lung tissues was not quantified in the current study. Finally, viral load was assessed based on viral genome copy numbers, without testing infectious virus titers. The viral nucleic acids detected by qRT-PCR include defective viral fragments and non-infectious viral particles, which cannot fully reflect the actual number of viable infectious virus particles, thereby limiting the accuracy of our viral load data. Future studies will include transcriptome analyses at multiple time points, combined with dual verification at the mRNA and protein levels, in vivo lung viral burden detection, and more accurate infectious virus quantification, to thoroughly analyze CPS-mediated virus–host immune interactions.

In summary, H9N2 IAV and *K. pneumoniae* co-infection can trigger severe lung damage by activating multiple innate immune pathways. *K. pneumoniae* CPS reshapes the lung immune microenvironment, amplifies inflammatory damage, and worsens cell apoptosis, jointly regulating inflammatory pathology and viral replication, and playing a key role in co-infection pathogenesis. This study provides important experimental evidence for understanding the pathogenic mechanism of H9N2 IAV secondary to *K. pneumoniae* infection.

## 5. Conclusions

Co-infections of H9N2 IAV and *K. pneumoniae* altered gene expression in the lungs of the experimental mice, disrupting internal homeostasis. The differentially expressed genes were associated with immune responses, cell proliferation, and apoptosis, and were involved in the TLR, RLR, TNF, and NLRP3 signaling pathways. H9N2 IAV and *K. pneumoniae* co-infection exacerbated inflammatory responses, worsened cell viability, aggravated apoptosis in lung epithelial cells, and indirectly reduced H9N2 IAV replication.

## Data Availability

The original contributions presented in this study are included in the article.

## Abbreviations

The following abbreviations are used in this manuscript:

CFU	Colony-forming unit
dpi	Day post-infection
EID50	50% egg infective dose
HE	Hematoxylin and eosin
hpi	Hour post-infection
*K. pneumoniae*	*Klebsiella pneumoniae*
IAV	Influenza A virus
